# Predictive value of background parenchymal enhancement on breast magnetic resonance imaging for pathological tumor response to neoadjuvant chemotherapy in breast cancers: a systematic review

**DOI:** 10.1186/s40644-024-00672-0

**Published:** 2024-03-11

**Authors:** Xue Li, Fuhua Yan

**Affiliations:** 1 Department of Radiology, Ruijin Hospital, Shanghai Jiao Tong University School of Medicine, No. 197 Ruijin Er Road, Shanghai, 200025 China; 2 Department of Radiology, Beijing Hospital, National Center of Gerontology, Institute of Geriatric Medicine, Chinese Academy of Medical Sciences, No. 1 DaHua Road, Dong Dan, Beijing, 100730 PR China; 3 Graduate School of Peking, Union Medical College, Beijing, PR China

**Keywords:** Breast cancer, Machine learning, Pathologic complete response, Neoadjuvant therapy, MRI radiomics

## Abstract

**Objectives:**

This review aimed to assess the predictive value of background parenchymal enhancement (BPE) on breast magnetic resonance imaging (MRI) as an imaging biomarker for pathologic complete response (pCR) after neoadjuvant chemotherapy (NACT).

**Methods:**

Two reviewers independently performed a systemic literature search using the PubMed, MEDLINE, and Embase databases for studies published up to 11 June 2022. Data from relevant articles were extracted to assess the relationship between BPE and pCR.

**Results:**

This systematic review included 13 studies with extensive heterogeneity in population characteristics, MRI follow-up points, MRI protocol, NACT protocol, pCR definition, and BPE assessment. Baseline BPE levels were not associated with pCR, except in 1 study that reported higher baseline BPE of the younger participants (< 55 years) in the pCR group than the non-pCR group. A total of 5 studies qualitatively assessed BPE levels and indicated a correlation between reduced BPE after NACT and pCR; however, among the studies that quantitatively measured BPE, the same association was observed only in the subgroup analysis of 2 articles that assessed the status of hormone receptor and human epidermal growth factor receptor 2. In addition, the predictive ability of early BPE changes for pCR was reported in several articles and remains controversial.

**Conclusions:**

Changes in BPE may be a promising imaging biomarker for predicting pCR in breast cancer. Because current studies remain insufficient, particularly those that quantitatively measure BPE, prospective and multicenter large-sample studies are needed to confirm this relationship.

**Supplementary Information:**

The online version contains supplementary material available at 10.1186/s40644-024-00672-0.

## Advances in knowledge

1. The extensive heterogeneity of studies in population characteristics, MRI follow-up points, MRI protocol, NACT protocol, pCR definition, and BPE assessment impedes the consistency of study findings and the efficient clinical application of BPE.
2. The total reduction in visually assessed BPE after NACT is associated with pCR. Since baseline BPE may not predict pCR in breast cancer, further studies with subgroup analyses should be performed based on age, HR, and HER2 status.
3. The predictive value of early reduction of quantitatively assessed BPE following NACT for pCR remains controversial, requiring prospective, multicenter large-sample studies to confirm this relationship.

## Introduction

In the 1990s, two clinical trials (protocols B-18 [1] and B-27 [2]) of the National Surgical Adjuvant Breast and Bowel Project (NSABP) reported efficacy and clinical value for preoperative neoadjuvant chemotherapy (NACT) in breast cancer, triggering a paradigm shift in breast cancer treatment [3]. Today, NACT is increasingly used in breast cancer treatment and aims at downstaging cancer and turning unresectable lesions into resectable ones, which increases patient breast conservation rates [4, 5]. Compared with postoperative chemotherapy, NACT allows early detection and evaluation of tumor treatment responses and timely optimization of inefficient treatment protocols, reducing unnecessary toxicity [5, 6]. In addition, pathologic complete response (pCR) of breast cancer after NACT is highly associated with increased patient survival and is considered the most important surrogate endpoint for predicting long-term outcomes [7, 8]. However, because pCR is determined only by collecting a surgically resected breast tumor specimen, introducing biomarkers to estimate tumor response to NACT before surgery is of utmost clinical significance.

Background parenchymal enhancement (BPE) is the contrast enhancement of fibroglandular tissue on dynamic contrast-enhanced magnetic resonance imaging (DCE-MRI). It has become the point of discussion on imaging biomarkers for predicting breast cancer risks and treatment outcomes, including tumor response [9]. It is an individual-specific characteristic that can vary over time and is influenced by age, menstrual cycle phase, and hormonal therapy [9]. Moreover, evidence suggests higher BPE is associated with higher basal metabolic activity of the normal breast tissue [10]. Current research assesses BPE by qualitative evaluation or fully or semi-automated quantification. The qualitative evaluation typically utilizes the Breast Imaging Reporting and Data System (BI-RADS) lexicon that classifies BPE into 4 qualitative categories (minimal, mild, moderate, and marked) and is performed by individual radiologists. The quantitative evaluation uses computerized segmentation algorithms (e.g., fuzzy c-means clustering algorithm) to fully or semi-automatically segment breast fibroglandular tissue and quantitatively calculate BPE to minimize subjective assessment [9].

Concerning the impact of BPE on breast cancer diagnosis and treatment, relevant studies have shown that moderate or marked BPE do not affect the detection rate of breast cancer but increase call-back rate [11]. In addition, they impact the assessment accuracy of tumor sizes, leading to positive margins after surgery [12]. Furthermore, a meta-analysis confirmed that higher BPE levels are associated with higher rates of breast cancer in high-risk women [13]. Although numerous studies have evaluated the relationship between qualitatively or quantitatively assessed BPE levels and tumor response to neoadjuvant therapy, the findings remain conflicting [14–26].

Therefore, this study aimed to systematically review the current literature to evaluate the predictive value of BPE on breast MRI as an imaging biomarker for pCR after NACT.

## Materials and methods

### Literature search and study selection

A comprehensive search in the PubMed, MEDLINE, and Embase databases was conducted for studies published until June 11, 2022. A combination of “background parenchymal enhancement,” “magnetic resonance imaging,” “neoadjuvant therapy,” and “breast” synonyms was used (see Supplementary Material for details) as keywords to identify potentially eligible articles for this review.

Studies were included if they

- ▪ reported the predictive value of BPE assessed on DCE-MRI for tumor response after NACT for breast cancer
- ▪ were original studies published in English

The studies were excluded if they

- ▪ were reviews, meta-analyses, conference abstracts, case reports, letters, and editorials
- ▪ studies about other treatments (e.g., neoadjuvant endocrine therapy)
- ▪ studies that did not discuss tumor response after neoadjuvant chemotherapy
- ▪ studies that were unavailable as full texts
- ▪ studies unavailable in English

The study selection process contained 2 steps. First, 2 reviewers independently screened the titles and abstracts according to the inclusion and exclusion criteria to ensure the relevance of the included articles. Second, the reviewers jointly screened the full texts of the selected articles to evaluate their eligibility. All disagreements were resolved by discussion and reaching a consensus between both reviewers.

### Data extraction and data analysis

The data extracted from the included studies were as follows:

- ▪ study characteristics (name of the first author, publication year, and study design)
- ▪ population characteristics (number of patients, mean age, menopausal status, tumor type, and pCR rate)
- ▪ DCE-MRI characteristics (e.g., scan protocol and sequence parameters)
- ▪ NACT treatment protocol, including treatment cycles and MRI follow-up time points
- ▪ BPE assessment methods (e.g., qualitative or quantitative assessments, DCE-MRI phases used for BPE assessment, and their corresponding calculation formulae)
- ▪ pCR definition, number of readers, and intra- and inter-reader variabilities for qualitative BPE assessment

Because some terms across different publications were highly variable, those expressed differently but having the same concept were standardized to facilitate readability.

### Quality assessment

The quality of the included studies was assessed using the Quality Assessment of Diagnostic Accuracy Studies-2 (QUADAS-2) tool [27] and Review Manager (v. 5.4) software. The risk of bias and applicability of the included articles were graded based on 4 domains: patient selection, index tests, reference standards, flow and timing.

## Results

### Study selection

**Fig. 1:**
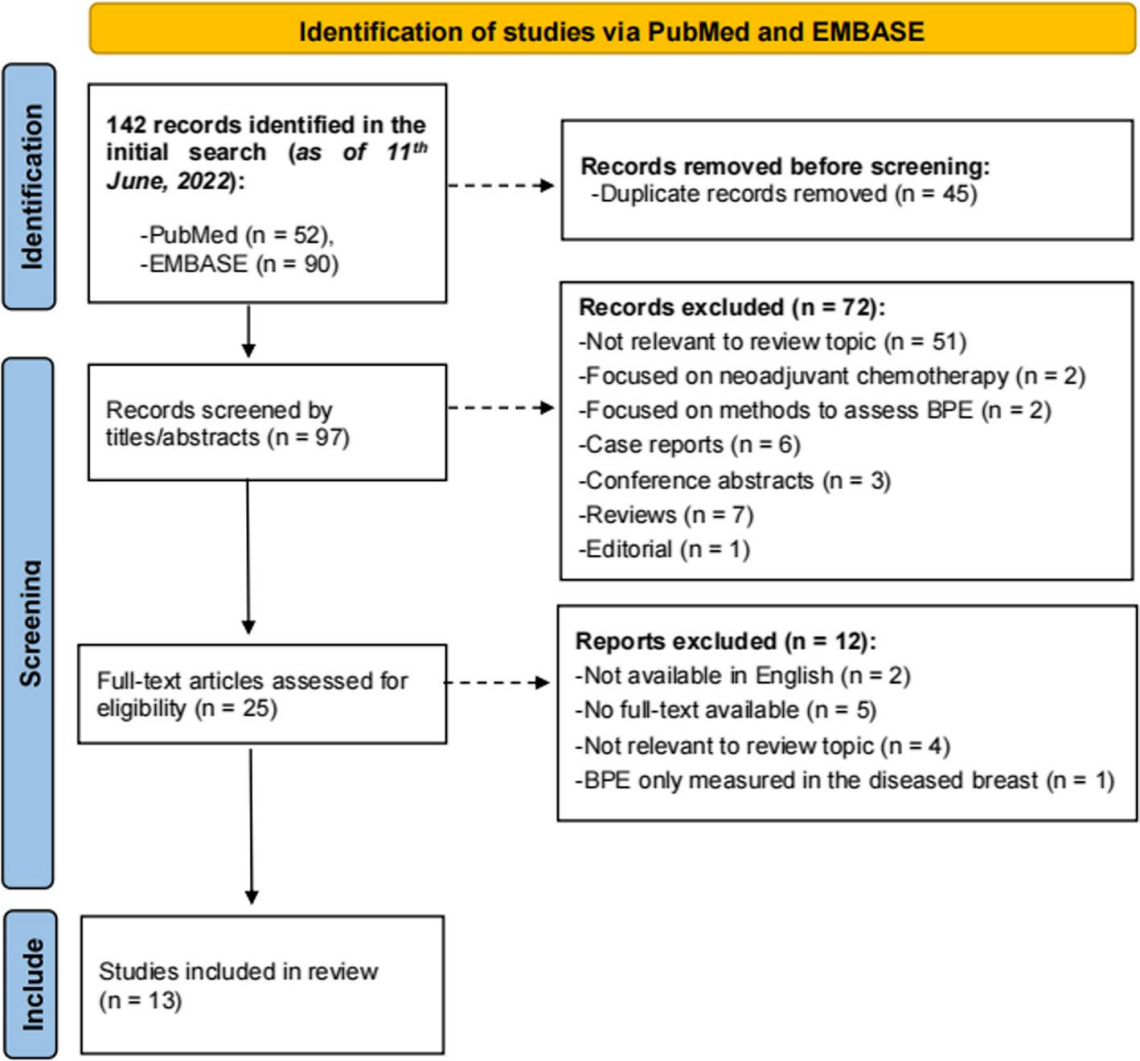
Flow diagram of the selection process for the included studies

A total of 142 records were identified through electronic database searches (52 records from PubMed and 90 from Embase), and 45 duplicate articles were removed. The titles and abstracts of the remaining 97 articles were assessed, of which 25 were deemed relevant. Among the relevant articles, 5 were excluded due to full-text unavailability, and 7 were considered ineligible for inclusion. The remaining 13 articles were included for the final analysis (Fig. 1).

### Risk of bias assessment

**Fig. 2:**
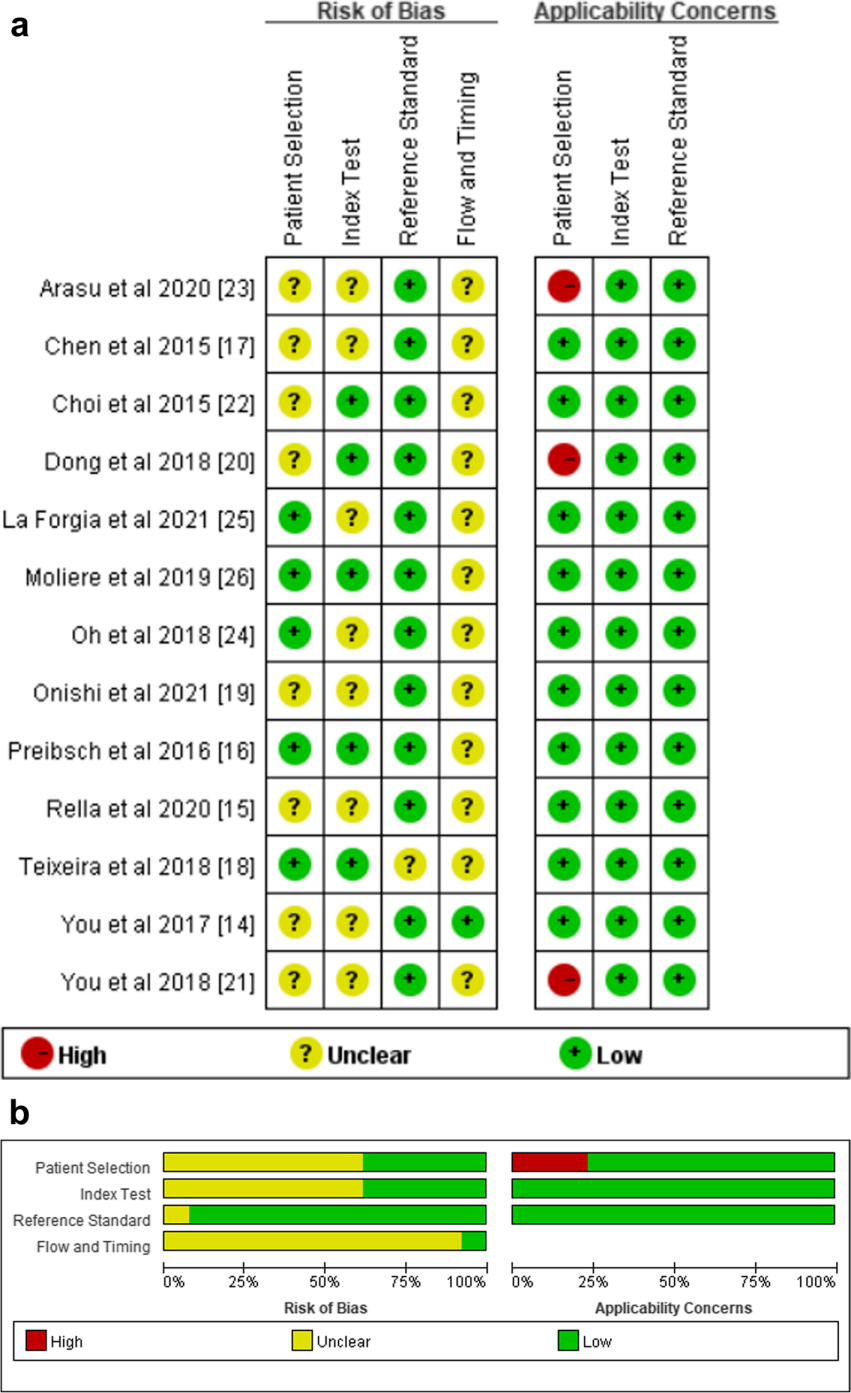
The risk of bias and applicability concerns graph (**a**) and summary (**b**) assessed for each included article using the QUADAS-2 tool

Figure 2 displays the QUADAS-2 evaluation of the risk of bias and applicability concerns in the 13 studies included in the systematic review.

In the patient selection domain, the risk of bias was unclear for 8 studies [14, 15, 17, 19–23] since they did not state whether the patients were consecutive. Similarly, the risk of bias in the index test domain was unclear in 8 studies [14, 15, 17, 19, 21, 23–25] as they did not indicate whether the BPE assessment was performed without knowing the pCR results. In the reference standard domain, the risk of bias was unclear in 1 study [18] owing to undefined pCR. Finally, in the flow and timing domain, it was unclear for nearly all studies since no specific period was defined between the MRI and the surgery or biopsy. In addition, 3 studies [20, 21, 23] had high applicability concerns due to the primary inclusion of patients with epidermal growth factor receptor 2 (HER2)-positive or -negative breast cancer.

### Study and patient characteristics

Among the 13 included studies, 2 [19, 23] were multicenter studies (with 1 prospective), 1 [18] was a retrospective dual-center study, 1 [22] did not specify the study type, and the rest were retrospective single-center studies (Table 1). The sample size of the 13 studies ranged from 46 [17] to 882 [19] patients, and their mean age was between 45 and 50 years.

**Table 1:** Study and patient characteristics

Study ID	Study design	Study population	Mean age ± SD, age range (years)	Pre-menopausal status / total population	Tumor histological type	NAC Protocol (*n* = number of patients)	pCR rate	Treatment response definition
You et al. 2017 [14]	Retrospective study; Single center	90 patients with unilateral breast cancer	49.84 ± 10.04 years, 28 – 69 years	50/90; pCR group: 13/25; non-pCR group: 37/65	72 IDC, 5 DCIS, 2 ILC and 11 tumor classification unclear	CEF (*n* = 5), PC (*n* = 20), PE (*n* = 2), (*n* = 29) and PCH (*n* = 39); 6 or 8 cycles	27.78%	pCR or non-pCR; The absence of invasive carcinoma (residual DCIS allowed) by pathologic examination
Rella et al. 2020 [15]	Retrospective study; Single center	228 patients with unilateral breast cancer	47.6 ± 10 years, 24 – 74 years	pCR group: 18/30; non-pCR group: 121/198	162 IDC, 29 ILC and 37 other invasive carcinomas	A combination of doxorubicin and cyclophosphamide (2—4 cycles) and taxanes (+ trastuzumab if HER2-positive)	13.2%	pCR or non-pCR; The absence of residual invasive cancer cells in the breast and ipsilateral lymph nodes (DCIS may have been present) (ypT0/is, ypN0)
Preibsch et al. 2016 [16]	Retrospective study; Single center	73 patients with 80 biopsy-proven breast cancers	48.5 ± 9.9 years; 26.8 – 71.2 years	NR	71 IDC, 8 ILC and 1 invasive apocrine carcinoma	EC + docetaxel (*n* = 75), trastuzumab and zoledronic acid (*n* = 1), paclitaxel and avastin (*n* = 1), taxol and herceptin (*n* = 2) and vinorelbine and trastuzumab (*n* = 1)	CR (19%); PR (57%)	According to RECIST 1.1 criteria; CR (the disappearance of all target lesions was reached); PR (at least 30% decrease in the sum of the diameters of target lesions in comparison with the baseline sum diameters); SD and PD
Onishi et al. 2021 [19]	Retrospective study; Multiple center	882 patients with unilateral breast cancer (with breast cancer at high risk for early recurrence)	48 ± 10 years; NR	457/882	NR	Paclitaxel and/or a combination of 9 experimental agents (12 cycles), followed by anthracycline-cyclophosphamide (4 cycles; + trastuzumab if HER2-positive)	33%	pCR or non-pCR; The absence of residual invasive carcinoma in the breast and axillary lymph nodes after NAC
Chen et al. 2015 [17]	Retrospective study; Single center	46 patients with unilateral breast cancer	50 ± 11 years; NR	NR	40 IDC, 5 ILC and 1 mixed IDC and ILC	Dose-dense AC (2—4 cycles) followed by taxane regimen (After the patient received 2 cycles of AC, the oncologist determined the response and decided the next regimen)	52%	pCR or non-pCR; The absence of malignant cancer cells
Dong et al. 2018 [20]	Retrospective study; Single center	51 patients with HER2-positive unilateral IDC	46.24 ± 8.79 years; 27 – 63 years	27/51; pCR group: 14/23; non-pCR group: 13/28	All were HER2-positive IDC	Docetaxel, carboplatin, and trastuzumab (*n* = 29); doxorubicin, cyclophosphamide, docetaxel, and trastuzumab (*n* = 22); at least 6 or 8 cycles	45.1%	pCR or non-pCR; No residual tumor or only DCIS
You et al. 2018 [21]	Retrospective study; Single center	71 patients with HER2-positive unilateral breast cancer	47.65 ± 10.10 years; 26 – 71 years	37/71	70 IDC, 1 ILC	4 cycles of paclitaxel, carboplatin in combination, and trastuzumab	47.89%	pCR or non-pCR; The absence of residual invasive cancer upon hematoxylin and eosin evaluation of a complete resected breast specimen and all sampled regional lymph nodes following completion of neoadjuvant systemic therapy
Choi et al. 2015 [22]	NR	98 patients with invasive breast carcinoma (1 patient with bilateral breast cancer)	50 years; 29 – 81 years	NR	76 IDC; 3 ILC; 1 invasive carcinoma with signet ring cell, 1 IMPC, 8 DCIS, 1 LCIS, and 1 sclerosing adenosis (no tumors remained in 7 cases)	AT (*n* = 47); AC (*n* = 13); C-LTZ (*n* = 1); C-EPRV (*n* = 1); C-CPV (*n* = 1); EP (*n* = 3); EC (*n* = 15); ET (*n* = 17); DP (*n* = 1); 4 or 6 cycles	17.3%	pCR or non-pCR; The absence of microscopic residual tumor or invasive foci but the presence of DCIS
Arasu et al. 2020 [23]	Prospective study; Multiple center	88 patients with HER2-negative stage II or III breast cancer	pCR: 46.9 years; non-pCR: 48.8 years	pCR group: 20/29; non-pCR group: 35/59	NR	Paclitaxel alone or in combination with experimental NAC agents for 12 cycles, followed by 4 cycles of AC	33.0%	pCR or non-pCR; The absence of residual invasive cancer in the breast or lymph nodes at the time of surgery
Oh et al. 2018 [24]	Retrospective study; Single center	186 patients with invasive breast cancer	45 years; 25 – 81 years	128/186	NR	Adriamycin with cyclophosphamide plus docetaxel (*n* = 126); HER2 targeted agent-based regimens (*n* = 22); adriamycin with cyclophosphamide (*n* = 20); and FEC (*n* = 18)	20.4%	pCR or non-pCR; The absence of residual invasive cancer cells in the breast and ipsilateral lymph nodes (DCIS may have been present) (ypT0/is, ypN0)
La Forgia et al. 2021 [25]	Retrospective study; Single center	80 patients with breast cancer from stage I to stage II	49 years; 31 – 80 years	32/80	NR	4 cycles of anthracycline combined with cyclophosphamide, and only taxanes (with and without anti-HER2-positive therapy) in the following 3 months	CR (15%); PR (55%)	According to RECIST 1.1 criteria; CR (Disappearance of all target lesions; Any pathological lymph nodes must have reduction in short axis to < 10 mm); PR (At least a 30% decrease in the sum of diameters of target lesions, taking as reference the baseline sum diameters); SD and PD
Teixeira et al. 2018 [18]	Retrospect cross-sectional observational study; Dual center	150 patients with unilateral invasive breast cancer	45.2 years; 20 – 74 years	NR	NR	NR	NR	pCR or non-pCR; NR
Moliere et al. 2019 [26]	Retrospective study; Single center	102 patients with breast cancer	49.8 years; NR	54/102	NR	FEC (*n* = 84), additional weekly taxanes (*n* = 51) and trastuzumab (*n* = 33)	26.5%	pCR or non-pCR; The absence of residual invasive tumor in both the breast and the axillary nodes

*NR* note reported

*Abbreviations*: *IDC* Invasive ductal carcinomas, *DCIS* Ductal carcinoma in situ, *ILC* Invasive lobular carcinoma, *CR* Complete remission, *PR* Partial response, *SD* Stable disease, *PD* Progressive disease, *pCR* Pathologic complete response, *IMPC* Invasive micropapillary carcinoma, *LCIS* Lobular carcinoma in situ, *HER2* Human epidermal growth factor receptor 2, *NAC* Neoadjuvant chemotherapy

Significant heterogeneity was found in tumor histological type reported in 7 studies. Choi et al. [22] reported 7 histological types of breast cancer, whereas Dong et al. [20] included only HER2-positive invasive ductal carcinoma. The NACT protocol, including chemotherapeutic agents and treatment cycles, also varied considerably among the studies. In addition, tumor response assessment following chemotherapy was not uniform across the studies, and most breast cancer outcomes were categorized as pCR or non-pCR. Nonetheless, La Forgia et al. [25] and Preibsch et al. [16] assessed 4 possible outcomes according to the RECIST 1.1 criteria [28]: complete remission (rates 19% or 15%), partial response, stable disease, and progressive disease. The pCR rates fluctuated between 13.2% and 52.0% in 10 studies [14, 15, 17, 19–24, 26], and the pCR definition in these studies was not uniform.

### MRI characteristics

High heterogeneity was observed across 5 aspects: field strength, manufacturer, dosage, injection rate, and DCE acquisition and sequence parameters (Table 2). Among the 13 included studies, 9 used 1.5-T MRI [14–18, 21, 24–26], 3 used 1.5-T and 3.0-T [19, 22, 23], and only 1 used 3.0-T [20]. Among the 10 articles that disclosed dosage information, the majority administered 0.2 mL/kg or 0.1 mmol/kg [15, 17, 18, 20, 23–26], while only 1 gave 0.16 mmol/kg [16] and 0.2 mmol/kg [22] each. In addition, these studies maintained the injection rate between 2 mL/s and 2.5 mL/s. Furthermore, DCE-MRI mostly contained 1 pre-contrast and 3 to 12 post-contrast acquisitions, except for 2 studies that did not report specific information [19, 23], and 1 study [17] acquired 4 pre-contrast and 7 post-contrast images. Additionally, the DCE acquisition mentioned above used different acquisition time points or temporal resolution, as observed in Table 2.

**Table 2:** Detailed parameters of MRI protocol in the included articles

Study ID	Field strength	Manufacturer	Dosage	Injection rate	The name and vendor of contrast agent	Spatial resolution	DCE phase	DCE acquisition time points or temporal resolution
You et al. 2017 [14]	1.5-T	Aurora Imaging Technology, Aurora Systems, Inc., Canada	NR	2 mL / s	NR	In-plane spatial resolution = 1 mm	1 pre- and 4 post-contrast acquisitions	At 90, 180, 270, and 360 s after injection
Rella et al. 2020 [15]	1.5-T	Signa® Excite; GE-medical system	0.2 mL / kg	2 mL / s	Gadobenate dimeglumine; MultiHance®, Bracco)	Pixel size = 0.7 mm^2^	1 pre- and 5 post-contrast acquisitions	NR
Preibsch et al. 2016 [16]	1.5-T	Philips Achieva, Hamburg, Germany	0.16 mmol / kg	NR	Gadobutrol; Gadovist®, Bayer HealthCare AG, Berlin, Germany	In-plane spatial resolution = 0.8 mm	1 pre- and 7 post-contrast acquisitions	NR (post-contrast images obtained at ~ 150 s were used for BPE analysis)
Onishi et al. 2021 [19]	1.5-T or 3.0-T	NR	NR	2 mL / s	NR	In-plane spatial resolution ≤ 1.4 × 1.4 mm^2^	1 pre- and multiple post-contrast acquisitions	80–100 s per dynamic acquisition
Chen et al. 2015 [17]	1.5-T	Philips Medical System, Best, Netherlands	0.1 mmol / kg	NR	Omniscan; Nycomed-Amersham, Princeton, NJ	In-plane spatial resolution = (1.3–1.5) × (2.5–3.0) mm^2^	4 pre- and 12 post-contrast acquisitions	42 s per dynamic acquisition
Dong et al. 2018 [20]	3.0-T	Skyra, Siemens, Munich, Germany	0.2 mL / kg	2.5 mL / s	Gadopentetate dimeglumine; Germany, Berlin, Schering Co, Ltd (SCHERING)	In-plane spatial resolution = 1.1 × 0.8 mm^2^	1 pre- and 7 post-contrast acquisitions	NR
You et al. 2018 [21]	1.5-T	Aurora Imaging Technology, Aurora Systems, Inc., Vancouver, Canada	NR	2 mL / s	Magnevist; Bayer Schering Pharma, Berlin, Germany	In-plane spatial resolution = 1 mm	1 pre- and 4 post-contrast acquisitions	At 90, 180, 270, and 360 s after injection
Choi et al. 2015 [22]	1.5-T or 3.0-T	Achieva; Philips Medical Systems, Best, The Netherlands / Magnetom Verio; Siemens Medical Solutions, Erlangen, Germany	0.2 mmol / kg; 0.1 mmol / kg	NR	Gadovist; Bayer Schering Pharma AG	NR	1 pre- and 6 post-contrast acquisitions	At 7, 67, 127, 187, 247, and 367 s after injection
Arasu et al. 2020 [23]	1.5-T or 3.0-T	NR	0.1 mmol / kg	2 mL / s	NR	In-plane spatial resolution ≤ 1.4 mm	Pre- and multiple post-contrast images	Continued for at least 8 min after injection
Oh et al. 2018 [24]	1.5-T	Magnetom Avanto; Siemens Healthcare, Erlangen, Germany	0.1 mmol / kg; 0.2 mL / kg	2 mL / s	Dotarem; Guerbet, Roissy, France / Magnevist; Bayer Schering Pharma, Berlin, Germany	0.9 × 0.9 × 0.9 mm^3^	1 pre- and 5 post-contrast acquisitions	61 s per dynamic acquisition
La Forgia et al. 2021 [25]	1.5-T	Achieva, Philips Medical Systems, Best, The Netherlands	0.1 mmol / kg	2–2.5 mL / s	NR	1.5 × 1.5 × 1.5 mm^3^	1 pre- and 5 post-contrast acquisitions	60 s per dynamic acquisition
Teixeira et al. 2018 [18]	1.5-T	Achieva; Philips Medical Systems, Best, the Netherlands	0.1 mmol / kg	NR	NR	NR	1 pre- and 3 post-contrast acquisitions	NR
Moliere et al. 2019 [26]	1.5-T	Aera, Siemens Healthcare	0.1 mmol / kg	NR	Dotarem; Guerbet, Roissy, France	NR	1 pre- and 5 post-contrast acquisitions	90 s per dynamic acquisition; the late acquisition was centered at 6 min

*NR* Not reported

### BPE assessment methods

The reviewed pieces of literature were divided into 2 groups to assess the methods for BPE evaluation: studies with quantitative (*n* = 6; Table 3) [14, 15, 17, 19, 23, 26] or qualitative assessments (*n* = 8; Table 4) [16, 18, 20–22, 24–26]. For 1 article that used both methods to evaluate BPE, each was analyzed separately.

**Table 3:** Characteristics of studies with quantitative analysis of BPE levels

Study ID	Analysis form	ROI for BPE segmentation	Calculation formula for BPE levels	Calculation formula for change in BPE	DCE phases for BPE analysis	DCE acquisition time points or temporal resolution	MRI follow-up time points	No. of readers	Blindness to clinical data	Major findings
You et al. 2017 [14]	Fully automated	All the fifibroglandular tissue	BPE = (the enhanced fibroglandular tissue volume / total fibroglandular tissue volume) × 100%	ΔBPE1/2/3 = (BPE2nd/4th/6th follow-up MRI – BPEbaseline MRI) / BPEbaseline MRI *100%	The subtraction image of pre- and post-contrast MRI scans	At 90, 180, 270, and 360 s after injection	Before and after the 2nd, 4th, 6th, and 8th cycle of NAC	NR	NR	Reduction of BPE at the early stage of NAC was positively associated with pCR, especially in HR-negative status
Rella et al. 2020 [15]	Semi-automated	All the fifibroglandular tissue	BPE = [(SI_post–SI_pre) / SI_pre] × 100%	The enhancement rate identified in the MRI after chemotherapy minus the enhancement rate identifified at the baseline MRI (total BPE change)	The pre- and first post-contrast acquisitions	NR	Before and after NAC (*n* = 101); Before NAC, after the 4th cycle of NAC and after NAC (*n* = 127)	2	NR	In the subgroup of patients with stages 3 and 4 breast cancers and who were diagnosed with a HER2-negative tumor phenotype, a significant association was found between early BPE change and pCR (*P* = 0.020)
Onishi et al. 2021 [19]	Fully automated	The central 50% of axial sections in the breast	BPE = [(SI_early post – SI_pre) / SI_pre] × 100%	Evaluated as suppressed if ΔBPE was less than 0	The pre- and early post-contrast acquisitions	80–100 s per dynamic acquisition	Before treatment (T0), early treatment (3 weeks after treatment initiation, T1), interregimen (T2), and before surgery (T3)	1	NR	The association between BPE nonsuppression and lower pCR rate was detected at T2 and T3 in the HR-positive cohort
Chen et al. 2015 [17]	Computer-based segmentation algorithm	All the fifibroglandular tissue	BPEi = ((SI_posti–SI_pre)/ SI_pre) × 100%; BPE = ∑BPEi / 12	NR	4 pre- and 12 post-contrast acquisitions	42 s per dynamic acquisition	Before NAC; After 1st cycle or 2ed cycle of AC; After 4th cycle of AC or 2ed cycle of AC + 3 weekly second-line taxane-based regimen	NR	NR	Compared to baseline, BPE at F/U-1 was significantly decreased in the pCR group but not in the non-pCR group
Arasu et al. 2020 [23]	Semi-automated	All the fifibroglandular tissue	BPE = (SI_early post – SI_pre) / SI_pre	%ΔBPE0_1 = (BPE_1 – BPE_0)/BPE_0	Pre-contrast (time 0) and the first post-contrast acquisition (time 1)	Continued for at least 8 min after injection	Before NAC (T0), after 3 weeks of NAC, or early treatment (T1), after 12 weeks of therapy, or inter-regimen (T2), and after NAC and prior to surgery, (T3)	NR	NR	Among women with HER2-negative cancer, BPE alone demonstrated association with pCR in women with HR-positive HER2-negative breast cancer
Moliere et al. 2019 [26]	Fully automated	All the fifibroglandular tissue	BPE20% = V_BPE_ / V_FGT_ × 100%	ΔBPE20% = (BPE20%post—BPE20%pre) / BPE20%pre	3 subtraction images of pre- and the three post-contrast sequences, respectively	90 s per dynamic acquisition; The late acquisition was centered at 6 min	Before and after NAC	2	Blinded to pathology data	There was no signifcant diference between complete responders and non-complete responders in term of pre- and post-therapeutic BPE

*NR* Not reported

*Abbreviations*: *ROI* Region of interest, *HR* Hormone receptor, BPE Background parenchymal enhancement, *pCR* Pathologic complete response, *HER2* Human epidermal growth factor receptor 2, *SI* Signal strength, *NAC* Neoadjuvant chemotherapy

**Table 4:** Characteristics of studies with qualitative analysis of BPE levels

Study ID	BPE classification	Comparison Parameters	MRI sequence used	MRI follow-up time points	No. of readers	Blindness to clinical data	Inter- and intra-reader variability	Findings
Preibsch et al. 2016 [16]	Minimal (1), mild (2), moderate (3) and marked enhancement (4)	The baseline BPE; The change in BPE	Subtraction images	Before and after NAC	2	Aware that patients received NAC for breast cancer but blinded to results from another reader	Substantial (κ_right breast = 0.73, κ_left breast = 0.77) before NAC and moderate (κ_right = 0.43, κ_left = 0.60) after NAC; The change in BPE was moderate (κ_right = 0.62, κ_left = 0.60)	The decrease in BPE was significantly higher in the cases with CR than in those with PD (*P* = 0.02)
Dong et al. 2018 [20]	Minimal (1), mild (2), moderate (3) and marked enhancement (4)	BPE levels before and after NAC; The change in BPE	All images	Before and after NAC	2	Blinded to the patients’ information	κ = 0.773 at baseline; κ = 0.706 after NAC	The decrease in the BPE level was more dramatic in the pCR group than in the non-pCR group in the patients with HER2-positive breast cancer
You et al. 2018 [21]	Minimal (1), mild (2), moderate (3) and marked enhancement (4)	BPE levels before and in the 2nd NAC; The change in BPE	The early post-contrast images	Before and the 2ed cycle of NAC	2	NR	κ = 0.701 at baseline and κ = 0.784 after the 2nd NAC cycle	Decreased BPE after the 2nd NAC cycle was significantly associated with pCR
Choi et al. 2015 [22]	Minimal (1), mild (2), moderate (3) and marked enhancement (4)	BPE levels before and after NAC	Early post-contrast fat-suppressed T1W images or subtraction images	Before and after NAC	2	Retrospective analysis in a blinded manner without patient pathology data	NR	Minimal BPE was the most prominent after NAC, showing a similar rate between the pathologic responder group and the non-responder group
Oh et al. 2018 [24]	Minimal (1), mild (2), moderate (3) and marked enhancement (4)	BPE levels before and after NAC; The change in BPE	The first post-contrast sequences and maximum-intensity-projection images	Before and after NAC	2	NR	NR	Post-NAC BPE was lower in the pCR group than in the non-pCR group (*p* = 0.0004); Changes in BPE after NAC were significantly greater in the pCR group than in the non-pCR group
La Forgia et al. 2021 [25]	Minimal (1), mild (2), moderate (3) and marked enhancement (4)	BPE levels before and after NAC; BPE levels in intermediate; The change in BPE	The first post-contrast sequence	Before and after at least 3 months of NAC; After the NAC	3	NR	A good level of agreement with a Cohen’s kappa value of about 0.55 for each comparison	The response to therapy was found to be significantly associated with BPE reduction
Teixeira et al. 2018 [18]	Minimal, mild, moderate and marked enhancement	BPE levels before and after NAC; The change in BPE	The first contrast-enhanced image	Before and after the NAC	1	Blinded to all previous analyses	NR	A pre-NAC reduction in BPE, in the affected or contralateral breast, was an independent predictor of achieving a pCR
Moliere et al. 2019 [26]	Minimal (1), mild (2), moderate (3) and marked enhancement (4)	BPE levels before and after NAC	Subtracted enhanced series	Before and after NAC	2	Blinded to pathology data	Fair agreement between readers: κ = 0.44 for pre-therapeutic imaging and κ = 0.41 for post-therapeutic imaging; Fair to good intra-rater reliability (κ = 0.64 for pre-therapeutic imaging, κ = 0.50 for post-therapeutic imaging)	There was no significant difference between CR and non-CR in term of pre- and post-therapeutic BPE

*NR* Not reported

*Abbreviations*: *CR* Complete remission, *PD* Progressive disease, *pCR* Pathologic complete response, *NAC* Neoadjuvant chemotherapy, *BPE* Background parenchymal enhancement, *HER2* Human epidermal growth factor receptor 2

The quantitative assessment mainly involved fully automatic using fuzzy c-means clustering (*n* = 3) [14, 19, 26] or semi-automatic methods (*n* = 2) [15, 23] to segment the contralateral breast fibroglandular tissue from the region of interest (ROI) in the entire breast region. An exception was the study by Onishi et al. [19] that used the central 50% of axial images of the whole breast. The formula for the quantitative assessment varied across the studies, and the DCE phases used to assess BPE and MRI follow-up time points were also heterogeneous. The pre-contrast and first (or early) post-contrast acquisitions were the most commonly used to calculate BPE (*n* = 3) [15, 19, 23], with 5 out of 6 articles [14, 15, 17, 19, 23] reporting multiple MRI scan time points (i.e., pre-, mid-, and post-NACT).

The BI-RADS criteria for the qualitative analysis of BPE distinguishes BPE into 4 grades: minimal, mild, moderate, and marked. Most studies [16, 18–20, 22, 26] assessed only pre- and post-NACT BPE, whereas La Forgia et al. [25] assessed pre-, mid-, and post-NACT BPE. In 8 studies, BPE was evaluated by 1 to 3 readers, and each study used different evaluation methods (e.g., assessing both breasts and contralateral breast). In another 8 studies [16, 18, 20–22, 24–26], variations in the reported intraobserver and interobserver agreement were observed (κ = 0.41 to approx. 0.784). Only 5 articles disclosed whether the reader was blinded [16, 18, 20, 22, 26].

### Association between pCR and BPE at baseline or after NACT

A continuous decrease in BPE levels was observed at baseline and throughout NACT in most included articles.

At baseline, BPE levels showed no predictive value for tumor pathological response. This finding was reported in 8 articles (all, *P* > 0.05) [15–17, 20, 21, 24–26], while in the other 5 articles, the predictive value was not clearly stated.

Nevertheless, Chen et al. [17] performed a subgroup analysis based on patient age and observed significantly higher baseline BPE in younger patients (< 55 years) than in the older (≥ 55 years) (20.2% vs. 12.0%; *P* = 0.007). A sub-analysis of younger patients also showed higher baseline BPE in patients with pCR than those without (21.1% vs. 18.8%).

No significant difference in post-NACT BPE was observed between patients with or without pCR in 3 articles [15, 25, 26]. By contrast, Dong et al. [20] and Oh et al. [24] reported significantly lower post-NACT BPE in patients with pCR than those without pCR.

### Association between pCR and change in BPE

A total of 11 articles [14–21, 23–25] explored the association between the change in BPE levels and pCR, reporting promising results.

#### For qualitatively measured BPE

Among the articles that qualitatively measured BPE, 5 [16, 18–20, 25] explored the predictive role of change in BPE before and after NACT on tumor response, and 1 by Dong et al. [20] primarily focused on the HER2-positive population. These publications suggested that a reduction in post-treatment BPE compared with baseline BPE is predictive of pCR. Another study [21] included a population of HER2-positive patients and concluded that early reduction in BPE was associated with pCR.

#### For quantitatively measured BPE

Quantitative assessment of BPE often involved more than 2 MRI follow-up time points, and 5 papers [14, 15, 17, 19, 23] reported a relationship between the change in BPE levels at each time point and tumor response compared with baseline BPE. You et al. [14] measured BPE at 4 distinct time points (baseline and after the second, fourth, and sixth NACT cycles). They concluded that BPE reduction after the second NACT cycle showed the best diagnostic performance for pCR (AUC = 0.726). Chen et al. [17] also revealed a significant decrease in BPE at early stage (after 2 to 4 weeks of NACT) in patients with pCR compared with baseline BPE but not in patients without pCR. In further subgroup analyses, a similar BPE drop of the pCR group was observed only in younger (< 50 years) and estrogen receptor (ER)-negative patients.

Early BPE reduction may be a promising biomarker for predicting pCR in breast cancer, but this finding remains controversial. Rella et al. [15] reported no significant difference in early or total BPE change between patients with and without pCR. Further sub-analyses found that early BPE changes were significantly associated with pCR in women with stage III or IV breast cancer (*P* = 0.019) or in patients with stage III or IV tumors that restrained to HER2-negative breast cancer (*P* = 0.020).

Furthermore, the association between total BPE change and pCR was observed only in the subgroup analysis of 2 articles that assessed the status of hormone receptor (HR) and HER2.

A study [19] assigned a population of patients with breast cancer into HR-positive and HR-negative groups and revealed that non-suppressed BPE was associated with lower pCR rate at T2 (inter-regimen, *P* = 0.02) and T3 (before surgery, *P* = 0.003) in the HR-positive group. It performed further analyses based on menstruation and HER2 status and found that non-suppression of BPE at T3 was significantly associated with a lower pCR rate in perimenopausal, postmenopausal, and HER2-positive patients. In addition, Arasu et al. [23] focused on women with HER2-negative cancer and discovered that the BPE change from baseline to preoperation in the pCR group was significantly higher in the HR-positive subgroup than in the non-pCR group.

## Discussion

The major finding of this review was the high heterogeneity of the included studies in terms of study population characteristics, MRI follow-up time points, MRI protocol, NACT treatment protocol, pCR definition, and BPE assessment methods. Thus, only if the factors that contribute to high heterogeneity were identified could the predictive value of BPE for pCR after NACT be appropriately assessed. In conclusion, analysis of the included studies revealed that reduction in qualitatively assessed BPE levels after NACT is associated with improved tumor response in breast cancer. However, the potential of quantitatively assessed BPE changes as biomarkers for predicting pCR remains controversial.

Regarding the factors causing heterogeneity, more factors than mentioned in the conclusion of this review may also influence the assessment of the predictive value of BPE for pCR after NACT. Nonetheless, the factors focused on in this study are the most striking and should be standardized in the included studies.

The details of the BPE level assessments precisely illustrate the high variation in the analyzed studies. From the perspective of the evaluation method, BPE was determined by qualitative assessments according to the BI-RADS lexicon or quantitative measurements using fully-automated or semi-automated computerized protocols. In general, 1 to 3 readers first visually evaluated the qualitative BPE assessments, with inter- or intra-reader repeatability ranging from fair [26] to substantial [21]. The cause of the observed instability in repeatability is the subjectiveness of the visual assessment that relies on the reader's experience, considerably influencing BPE assessment. Moreover, unlike the computerized automatic assessment, the naked eye does not provide a precise or detailed evaluation of subtle changes in the BPE levels. Perhaps these points explain why almost all studies that qualitatively assessed BPE aimed to determine BPE alterations after NACT without focusing on BPE changes during NACT. The articles that quantitatively assessed BPE prioritized the change in BPE at several time points during NACT. Unlike subjectively evaluated BPE, the quantitative data is able to predict pCR early during NACT, assisting in the optimization of therapeutic pathways during NACT and reducing the economic burden on the healthcare system.

A critical step in quantitative BPE assessment is ROI selection. A few studies that predicted the outcome of patients with breast cancer after NACT chose ROIs focused on the tumor or the peri-tumor region [29]. Since the mammary gland is a symmetrical organ, and BPE is an intrinsic feature of breast parenchyma, most of the included literature quantitatively measured the contralateral breast and avoided tumor interference in ROI selection. However, quantitative methods for BPE were not standardized, as evidenced by the high heterogeneity among the studies [14, 15, 17, 19, 23, 26], including ROI for BPE segmentation (whole breast or the central 50% of axial sections of the breast), the formula for BPE quantification, the evaluation method of BPE changes (specific values or dichotomy), MRI protocol (such as contrast agent dose, injection rate, etc.), and selected DCE phase (pre- and early contrast images or subtraction images or all DCE phases). Because of this high variability, the data cannot be pooled for analysis, hindering the application of research insights to clinical practice. Therefore, having a unified and standardized quantitative method with easily accessible software is paramount.

Variation in tumor response definitions, NACT protocols (e.g., schemes, doses, and treatment cycles), tumor characteristics (e.g., size, ER, progesterone receptor, and HER2 status), and subtype distribution also contributes to the heterogeneity in the studies, which may be responsible for fluctuations in the pCR rates (13.2% to approx. 52.0%). For example, patients with breast cancers lacking estrogen or progesterone receptors have a 12-fold higher likelihood of achieving pCR [30]. In addition, the effect of different NACT protocols or NACTs on BPE should not be ignored. For example, a study reported that taxane-containing NACT was associated with almost complete suppression of BPE (average reduction, − 91.2% ± 7.5) [31]. Another study showed that BPE decreased early in tamoxifen treatment (< 90 days) and stayed unchanged significantly over a longer treatment time [32]. These findings imply that standardized NACT treatment protocols and stratification of breast cancer subtypes are imperative.

Analysis of the included studies revealed that BPE levels decreased during the treatment, which could be related to damage to normal tissue vessels by chemotherapeutic drugs, and a reduction in hormones due to chemotherapy-induced ovarian suppression. A study [33] stratified patients according to menstrual status and discovered younger women (< 55 years) exhibited higher baseline BPE and total BPE reduction than older women. The higher baseline BPE levels could be associated with higher estrogen levels in younger women and with hormone medication (e.g., hormonal contraception) and hormone therapy (e.g., hormone replacement therapy). In addition, an MRI performed at an inappropriate time of the menstrual cycle may also cause higher baseline BPE. More pronounced reductions in total BPE could be related to chemotherapy-induced ovarian suppression and higher baseline BPE levels. This review revealed BPE before NACT (i.e., baseline BPE) did not predict pCR. However, Chen et al. [17] found that after age stratification, the baseline BPE was higher in younger women (< 55 years) with pCR than in those from the same age group without pCR. This finding points to the need for more studies conducting stratification based on age, HR, or HER2 to evaluate whether baseline BPE predicts pCR.

The qualitative evaluation revealed that reduced BPE after NACT was associated with improved tumor response. This relationship could be a result of NACT drugs being delivered simultaneously to tumor and normal breast parenchyma via vascular perfusion, which kills tumor cells but also damages normal blood vessels. Although the studies that quantitatively assessed BPE offered varied results, most accepted that no association exists between total BPE changes and pCR, leaving the relationship between early BPE changes and pCR unresolved. Specifically, Chen et al. [17] and You et al. [14] suggested that reduction in early BPE was associated with pCR in breast cancer, particularly in HR- or ER-negative tumors. Rella et al. [15] did not confirm this claim but found the above association in patients with stage III or IV breast cancer and HER2-negative breast cancers in their sub-analysis. Hence, the disparity in these conclusions may again illustrate substantial heterogeneity in studies relying on quantitative BPE measurement.

### Study limitations and future outlook

In summary, this review analyzes many sources of heterogeneity that currently exist in studies focusing on breast BPE for predicting pCR. These factors greatly hinder the efficient clinical application of BPE. Quantitative measurement techniques for BPE assessment offer the possibility to monitor changes in BPE at various time points during NACT, providing a more objective approach than visual evaluation. However, the results of this systematic review indicate urgent attention to the standardization of MRI protocols (e.g., DCE acquisition and sequence parameters), usages of contrast agent, computerized segmentation methods, BPE quantitative calculation formula, unification of DCE phases selection, and MRI follow-up points to improve BPE assessment. Additionally, the MRI scan time (whether within the appropriate physiological cycle period) is critical for assessing BPE, whereas this information is rarely available from the included studies. Moreover, the findings of this study imply that menstrual status, NACT protocols, NACT cycles, HR, and HER2 status may also affect BPE and future perspectives should focus on dealing with these factors. Since over half of the studies included in our review had a sample size of fewer than 100 cases, and the sample size of further subgroup analysis in some studies was even more limited. Therefore, subgroup analyses could be performed based on each possible influencing factor after including a larger sample size. With the emergence of artificial intelligence in medical imaging, advanced techniques can be applied to standardize BPE measurement and construct diagnostic and predictive models for new treatment plans.

## Conclusion

In conclusion, Changes in BPE on breast MRI may have a predictive value for pCR in breast cancer NAC. However, current studies on this topic (especially those measuring BPE quantitatively) are still insufficient. Thus, future multicenter and prospective studies with larger sample sizes are required.

### Supplementary Information

**Supplementary Material 1.**

## Data Availability

Not applicable.

## Abbreviations

ACR: American College of Radiology
BI-RADS: Breast Imaging- Reporting and Data System
BPE: Background parenchymal enhancement
CR: Complete remission
DCE-MRI: Dynamic contrast-enhanced magnetic resonance imaging
ER: Estrogen receptor
HER2: Human epidermal growth factor receptor 2
HR: Hormone receptor
NACT: Neoadjuvant chemotherapy
NSABP: National Surgical Adjuvant Breast and Bowel Project
pCR: Pathologic complete response
QUADAS: Quality Assessment of Diagnostic Accuracy Studies
ROI: The region of interest
