# Supplementary material for: Predictive value of background parenchymal enhancement on breast magnetic resonance imaging for pathological tumor response to neoadjuvant chemotherapy in breast cancers: a systematic review
Source: Cancer Imaging. 2024 Mar 11;24:35. doi: 10.1186/s40644-024-00672-0 (PMC10926651; doi:10.1186/s40644-024-00672-0)
Supplement: Supplementary file 1 — Supplementary Material 1. [file 40644_2024_672_MOESM1_ESM.docx]

**Supplementary Material 1: Literature search strategy (up to 11^th^ June, 2022)**

**Search strategy for PubMed**

| **Search** | **Query** | **Results** |
| --- | --- | --- |
| **#1** | **Search: ((((((BPE) OR (background parenchymal enhancement)) OR (parenchymal enhancement)) OR (background PE)) OR (PE)) OR (parenchymal)) OR (fibroglandular enhancement)** | 108177 |
| **#2** | **Search: (((((magnetic resonance imaging) OR (MRI)) OR (Magnetic Resonance Images)) OR (magnetic imaging)) OR (contrast-enhanced MRI)) OR (dynamic contrast-enhanced MRI)** | 716276 |
| **#3** | **Search: (((((((((((((((((((((((Neoadjuvant Therapy) OR (Neoadjuvant Therapies)) OR (Neoadjuvant Treatment)) OR (Neoadjuvant Treatments)) OR (Neoadjuvant Radiotherapy)) OR (Neoadjuvant Radiotherapies)) OR (Neoadjuvant Radiation Treatment)) OR (Neoadjuvant Radiation Treatments)) OR (Neoadjuvant Radiation Therapy)) OR (Neoadjuvant Radiation Therapies)) OR (Neoadjuvant Radiation)) OR (Neoadjuvant Radiations)) OR (Neoadjuvant Chemotherapy)) OR (Neoadjuvant Chemotherapies)) OR (Neoadjuvant Chemotherapy Treatment)) OR (Neoadjuvant Chemotherapy Treatments)) OR (Neoadjuvant Chemoradiotherapy)) OR (Neoadjuvant Chemoradiotherapies)) OR (Neoadjuvant Chemoradiation Therapy)) OR (Neoadjuvant Chemoradiation Therapies)) OR (Neoadjuvant Chemoradiation Treatment)) OR (Neoadjuvant Chemoradiation Treatment)) OR (Neoadjuvant Chemoradiation)) OR (Neoadjuvant Chemoradiations)** | 49952 |
| **#4** | **Search: ((breast) OR (mammary)) OR (breasts)** | 633989 |
| **#5** | **#**1 AND #2 AND #3 AND #4 | 52 |

**Search strategy for Embase**

| **Search** | **Query** | **Results** |
| --- | --- | --- |
| **#1** | (BPE)/br OR (background parenchymal enhancement) OR (parenchymal enhancement) OR (background PE) OR (PE) OR (parenchymal) OR (fibroglandular enhancement) | 160241 |
| **#2** | (magnetic resonance imaging)/br OR (MRI) OR (Magnetic Resonance Images) OR (magnetic imaging) OR (contrast-enhanced MRI) OR (dynamic contrast-enhanced MRI) | 1235091 |
| **#3** | (Neoadjuvant Therapy)/br OR (Neoadjuvant Therapies) OR (Neoadjuvant Treatment) OR (Neoadjuvant Treatments) OR (Neoadjuvant Radiotherapy) OR (Neoadjuvant Radiotherapies) OR (Neoadjuvant Radiation Treatment) OR (Neoadjuvant Radiation Treatments) OR (Neoadjuvant Radiation Therapy) OR (Neoadjuvant Radiation Therapies) OR (Neoadjuvant Radiation) OR (Neoadjuvant Radiations) OR (Neoadjuvant Chemotherapy) OR (Neoadjuvant Chemotherapies) OR (Neoadjuvant Chemotherapy Treatment) OR (Neoadjuvant Chemotherapy Treatments) OR (Neoadjuvant Chemoradiotherapy) OR (Neoadjuvant Chemoradiotherapies) OR (Neoadjuvant Chemoradiation Therapy) OR (Neoadjuvant Chemoradiation Therapies) OR (Neoadjuvant Chemoradiation Treatment) OR (Neoadjuvant Chemoradiation Treatments) OR (Neoadjuvant Chemoradiation) OR (Neoadjuvant Chemoradiations) | 89183 |
| **#4** | (breast)/br OR (mammary) OR (breasts) | 957848 |
| **#5** | #1 AND #2 AND #3 AND #4 | 90 |
